# Associations Between Genetic Data and Quantitative Assessment of Normal Facial Asymmetry

**DOI:** 10.3389/fgene.2018.00659

**Published:** 2018-12-18

**Authors:** Sara Rolfe, Su-In Lee, Linda Shapiro

**Affiliations:** ^1^Center for Developmental Biology and Regenerative Medicine, Seattle Children's Research Institute, Seattle, WA, United States; ^2^Department of Genome Sciences, University of Washington, Seattle, WA, United States; ^3^Department of Computer Science, University of Washington, Seattle, WA, United States

**Keywords:** facial morphology, asymmetry, GWAS, 3DMD, feature extraction

## Abstract

Human facial asymmetry is due to a complex interaction of genetic and environmental factors. To identify genetic influences on facial asymmetry, we developed a method for automated scoring that summarizes local morphology features and their spatial distribution. A genome-wide association study using asymmetry scores from two local symmetry features was conducted and significant genetic associations were identified for one asymmetry feature, including genes thought to play a role in craniofacial disorders and development: *NFATC*1, *SOX*5, *NBAS*, and *TCF*7*L*1. These results provide evidence that normal variation in facial asymmetry may be impacted by common genetic variants and further motivate the development of automated summaries of complex phenotypes.

## 1. Introduction

The ability to make connections between genetic and phenotypic variation, hinges on phenotypic descriptions that are sufficiently detailed to capture the traits of interest. Biomedical imaging creates very high dimensional datasets that can be analyzed and used to extract phenotype descriptions. Traditional phenotyping from images consists of 2D and 3D measurements of landmarks manually placed on the image. Landmark data is typically sparse and is likely insufficient to capture the complexity necessary for an association with genetic data. A recent study, testing the relationship of facial asymmetry, estimated from nine mid-facial landmarks, with genetic variation at 102 single nucleotide polymorphism (SNP) loci, recently associated with facial shape variation, was unable to identify any SNP relating to asymmetry (Windhager et al., 2014). Methods for automatically phenotyping images and incorporating complex shape information, will be key in understanding the genetic basis of morphology. New approaches such as the BRIM method, developed by Claes et al., have shown the promise of summarizing morphological differences in novel ways to identify genes affecting normal morphology (Claes et al., 2014). The aim of this study is to use automated phenotyping to produce a score of facial asymmetry that incorporates local morphological measurements and their spatial distribution to investigate the genetic basis of facial asymmetry.

Previous analysis of symmetry in 3D facial images has included manual landmarks (Devlin et al., 2007; Stauber et al., 2008), automated measurements (Mercan et al., 2013, 2018), plane of symmetry calculation (Linden et al., 2017), and dense surface registration of a 3D image with a mirrored version (Yu et al., 2009; Demant et al., 2010; Darvann et al., 2011; Djordjevic et al., 2012). Surface registration-based methods show particular promise due to their independence from the plane of symmetry and ability to provide dense shape information across the surface of the face. Recent applications of surface-registration based methods have been validated by comparison to traditional landmark methods and used quantified asymmetry in individuals using the average transform magnitude or root mean squared error from predefined regions (Claes et al., 2011; Kornreich et al., 2016; Öwall et al., 2016; Verhoeven et al., 2016) and principle modes of variation (Lanche et al., 2007).

In previous work, our group have developed voxel-based deformable morphology analysis methods capable of quantifying facial development in embryos and postnatal animals, from 3D imaging modalities with high precision. Using compact feature representation of image differences, facilitates the comparisons between individuals and across groups (Rolfe et al., 2011, 2013, 2014). In this work we introduce a surface-registration based method to quantify bilateral symmetry in individuals and a metric to summarize how an individual's facial asymmetry and its spatial distribution compares to asymmetry in a healthy, control population.

In this study, we preform GWA analysis on two facial asymmetry scores using a sample of 3186 healthy subjects. Highly significant genetic associations were identified for one of our scores, including genes known to play a role in craniofacial disorders: *NFATC*1, *SOX*5, *NBAS* or likely to play a role in craniofacial development: *TCF*7*L*1.

## 2. Materials and Methods

### 2.1. Data

The datasets used in this work were previously collected as part of the FaceBase Consortium's 3D Facial Norms Dataset, described in detail by Weinberg et al. (2016). This study was one of the purposes, under informed consent, and IRB approval was obtained for their use in this work. The dataset consisted of 3D photographic facial surface scans and genetic data from 3186 3D facial meshes from healthy subjects of European Caucasian ancestry, between the ages of 3 and 40 years old. Error screening and quality control measures were followed to reduce variability, due to factors such as facial expression and poor image quality. Subjects were screened for many confounding environmental factors, including: (1) a personal history of facial trauma; (2) a personal history of facial reconstructive or plastic surgery; (3) a personal history of orthognathic/jaw surgery or jaw advancement; (4) a personal history of any facial prosthetics or implants; (5) a personal history of any palsy, stroke, or neurologic condition affecting the face; (6) a personal or family history of any facial anomaly or birth defect; and/or (7) a personal or family history of any syndrome or congenital condition known to affect the head and/or face (Weinberg et al., 2016). To demonstrate that the age range in this dataset did not disrupt the results, we also ran a GWAS excluding pre-pubertal individuals (under 14). The genes identified as significant on the whole dataset still met our threshold for significance on the restricted dataset. These results are reported in Figure S1 and Table S1.

All image data used in this project were acquired using the 3dMD imaging systems (3dMD, Atlanta, GA). These commercial stereo-photography systems incorporate multiple camera viewpoints to provide a 3D mesh of the human face, at no risk to the subject, with the high level of anatomical integrity required for medical research. Several recent studies have assessed the amount of noise or variability that may be present in 3D meshes acquired using the 3dMD system, compared to alternative methods such as direct anthropometry and digital photogrammetry (Dindaroğlu et al., 2016), or high-accuracy industrial “line-laser” scanning (Zhao et al., 2017). The findings from these studies suggest that the number of errors likely to be present in a 3dMD dataset is similar to, or an improvement over more traditional methods. The facial surface scans were stored as 3D meshes that were not aligned and may contain extraneous objects such as hair and clothing. Prior to analysis, images were preprocessed to remove noise, cropped to extract the facial region and aligned using custom software developed by our research group (Wu et al., 2014).

A standard set of 24 facial landmarks was collected for each 3D facial mesh. In this study, a subset of 18 landmarks was selected to minimize the number of subjects excluded, due to missing the landmark points. A diagram of the landmarks used for analysis is shown in Figure S2. Details on the procedures used to identify the landmarks on 3D facial surfaces can be found on the “Technical Notes” section of the 3DFN website (https://www.facebase.org/facial_norms/notes).

The genotype data consists of 964,193 SNPs on the Illumina (San Diego, CA) OmniExpress+Exome v1.2 array plus 4,322 custom SNPs chosen in regions of interest based on previous studies of the genetics of facial variation.

### 2.2. Facial Asymmetry Score

Most attempts to summarize image characteristics rely on global features that describe the image as a whole, or local features calculated point-wise across the image. Previous work evaluating asymmetry in facial images has tended toward a local, point-wise approach (Claes et al., 2011; Kornreich et al., 2016; Öwall et al., 2016). While these features have been shown to be effective, we propose a method to produce a richer phenotype description by scoring an individual's relationship to a model of normal asymmetry using both global and local differences. In this work, the global assessment of facial asymmetry is restricted to the region below the eyes (defined by the right and left endocathion landmarks). This restriction limits noise caused by eyelashes, eyebrows, and the hairline and is consistent with landmark-based analysis for this data set, as landmarks were not collected in the forehead region (Weinberg et al., 2016).

**Figure 1:**
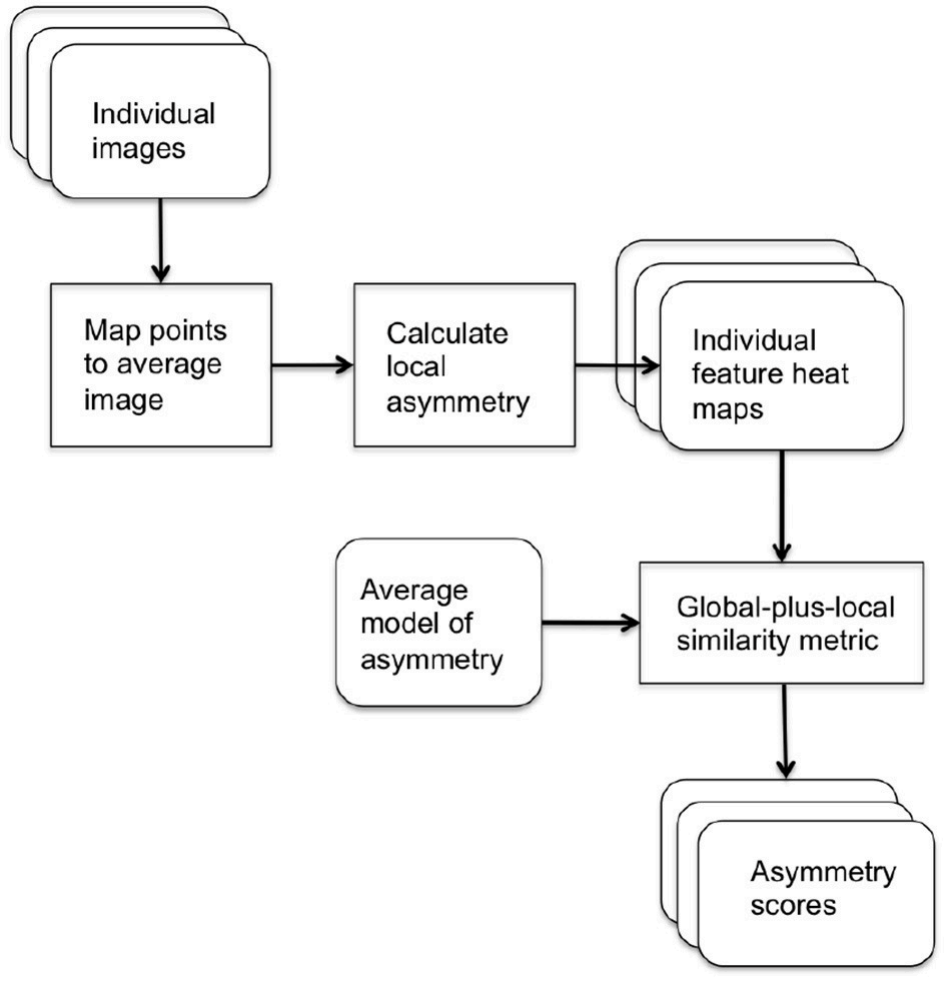
Block diagram of system for assigning asymmetry scores.

In our score assignment system, two local asymmetry metrics were defined to produce independent scores of asymmetry. The local metrics were assessed at each point on the surface of each image. For each asymmetry metric, a statistical model of asymmetry was calculated and each image was scored by its distance from the average model using our novel similarity measure to combine global distribution information with local point-wise correspondences, to produce a summary of the local and global differences. The block diagram of the asymmetry score assignment system is shown in Figure 1.

#### 2.2.1. Local Asymmetry Metrics

**Figure 2:**
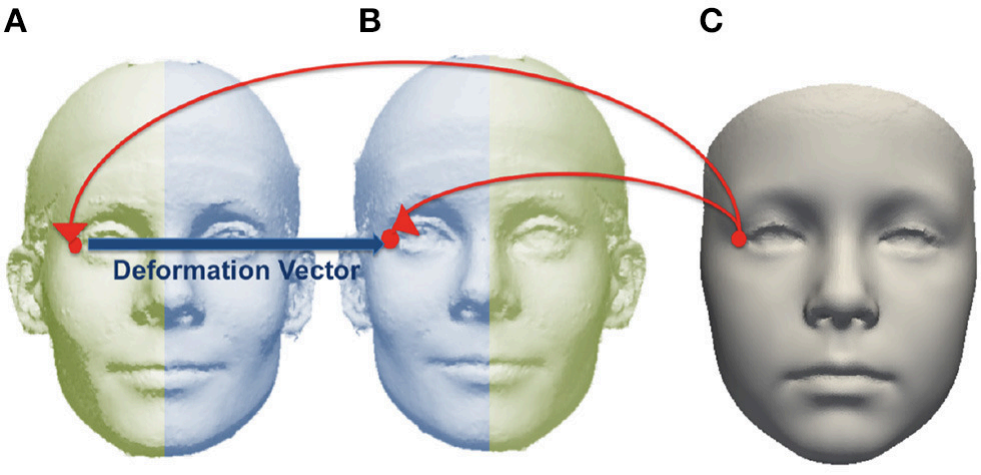
Example of corresponding point mapped from an average image **(C)** to subject mesh **(A)** and mirrored copy **(B)**. The asymmetry flow vector is defined between corresponding points on the subject mesh and its mirrored copy.

The 18 manually-placed landmarks shown in Figure S2 were used to align each subject mesh in a common orientation. After alignment, a base mesh was chosen and corresponding points in each source mesh were found for each point in the base mesh, using the dense point correspondence method developed by Hutton et al. (2003). The locations of corresponding points for each point in the base mesh were averaged over the group to generate the average mesh. Each subject mesh was mirrored across the mid-line and the original and mirrored image were densely mapped to the average mesh. For each point on the average image, an asymmetry flow vector was defined by the difference in position between the corresponding points on the mirror and original images, representing the transformation due to asymmetry. This is illustrated in Figure 2.

We defined two properties of local morphology calculated at each point on an individual facial mesh to capture independent aspects of facial asymmetry.

1. Angle of surface orientation: angle between the normal vectors at corresponding points on and the mirror image. This value quantifies the asymmetry in surface orientation at each point on the image.
2. Angle of deformation: angle between asymmetry flow vector and surface normal on the original image. This value quantifies the direction of the transformation between an image and its mirrored copy at each corresponding point.

**Figure 3:**
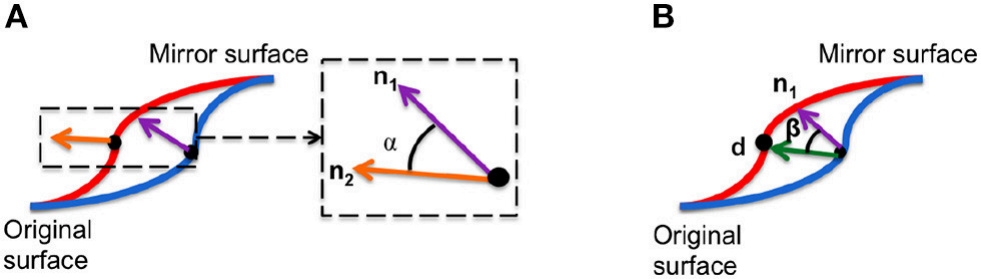
Two morphological features are used to densely characterize facial symmetry and averageness: **(A)** angle of surface orientation and **(B)** angle of deformation.

These local asymmetry features are illustrated in Figure 3. These angle-based features capture one aspect of asymmetry and are independent of the magnitude of asymmetry.

The magnitude of the deformation can also be used as a local feature of asymmetry using our method. It is defined at each point on an individual facial mesh as the length of the 3D vector between that point and the corresponding point on the mirror image. Results from this approach are included in Figure S3 and Table S5.

#### 2.2.2. Average Model of Normal Asymmetry

Some asymmetry is expected in normal human facial features and the type and amount expected varies with location on the face. For example, asymmetry in the corners of the lips and eyes is more common than asymmetry in the nasal tip. To take into account these spatial differences, each asymmetry score was based on the distance between an individual and an average model of normal asymmetry rather than the absolute asymmetry of the face.

**Figure 4:**
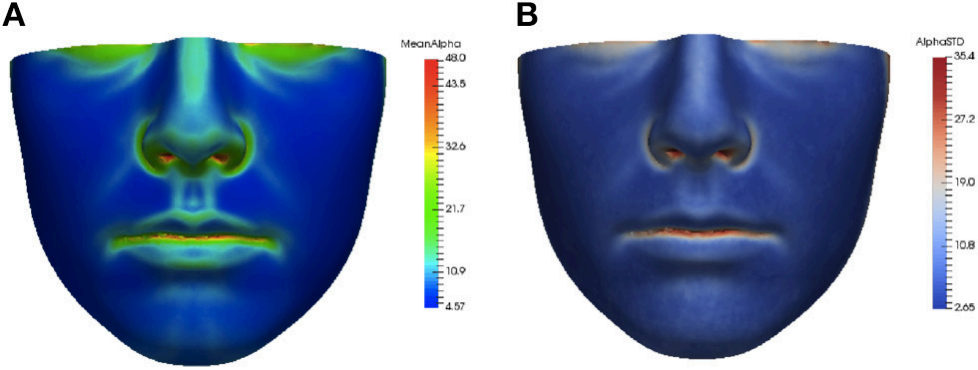
Average **(A)** and standard deviation **(B)** of angle of surface orientation feature for the data set.

The asymmetry heat maps were used to create an average model of normal asymmetry for each feature. For every point on the average mesh, the average and standard deviation of each feature distribution over all corresponding points in the dataset were calculated. The average and standard deviation heat maps for the angle of surface orientation feature are shown in Figure 4.

#### 2.2.3. Distance From Average Model of Normal Asymmetry

To assess the similarity between two feature heat maps the following questions must be addressed:

1. What feature values are present in the image?
2. Where are regions of similar feature values approximately located?

To simultaneously address these two questions, we developed a similarity metric that combines information about the global feature distribution and point-wise differences.

Histograms of image features provide a robust description of global image data that has proven to be powerful in detecting similarity. However, the use of histogram representations of features presents two primary drawbacks: the loss of spatial distribution information and the loss of information due to quantization. To address this, histograms can be augmented by the inclusion of additional spatial information and other local properties (Birchfield and Rangarajan, 2005; Lyons, 2009; Prabhu and Kumar, 2014; Zeng et al., 2015). In previous work, our group developed a method to simultaneously assess similarities in feature values and their regional distribution based on spatial histograms (Rolfe et al., 2014).

**Figure 5:**
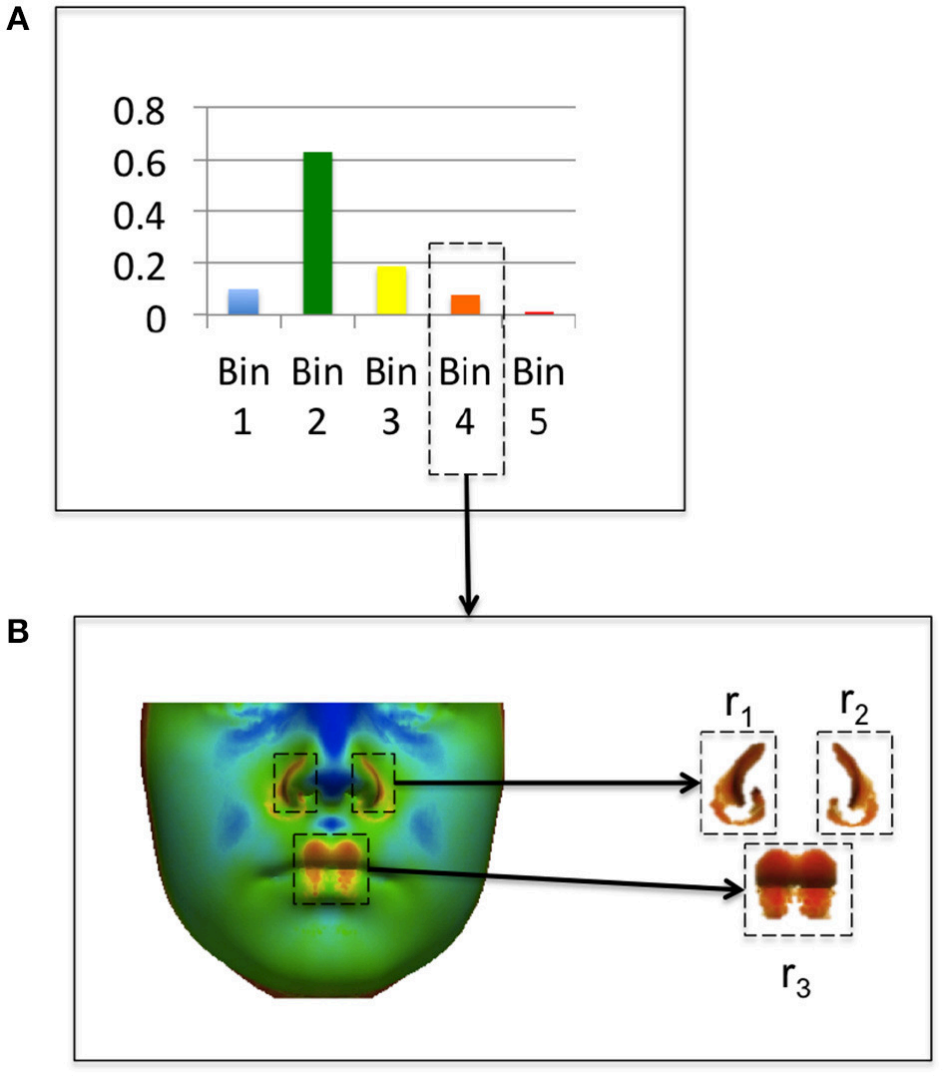
Example of coherent region extraction from a feature histogram bin. The feature histogram from an individual is shown in **(A)** and coherent regions in bin 4 are shown in **(B)**.

Intuitively, the spatial histogram, or spatiogram is an image histogram where the distribution of values is spatially weighted by the similarity of the spatial positions of the values in each bin. Typically, this is done by modeling the spatial location of the contents of each histogram bin with a single or mixture of Gaussian distributions. In this application the known point correspondences between images in the data set calculated in section 2.2.1 are leveraged to provide a more precise score of spatial matching between histogram bins. The spatial information is incorporated as the set of coherent feature regions in a histogram bin. For an image *I*, the histogram of *I* is defined as:

(1) $$\begin{array}{r} h_{I}\left(b\right)=n_{b},\;b=1,\ldots ,B, \end{array}$$

where *n*_*b*_ is the number of pixels with values assigned to the *b*-th bin and *B* is the total number of bins. Our spatially augmented histogram is defined as:

(2) $$\begin{array}{r} h\left(b\right)=<n_{b},R_{b}=\left(r_{b1},..,r_{bm}\right)>, \end{array}$$

where *n*_*b*_ is the number of points with values assigned to the bin *b*, and *R*_*b*_ is the set of *m* coherent regions *r*_*b*1_, …*r*_*bm*_ where *r*_*bi*_ is a vector of *j* point indexes < *x*_1_, …*x*_*j*_>. Coherence of regions is determined by computing connected components. A connected region *r*_*bi*_ is the set of mesh points such that for any point $x,x^{\prime }\in r_{bi}$, there is a path in *r*_*bi*_ to from *x* to *x*′. A threshold for coherence can be set for a connected region with greater than τ mesh points. For this study regions with τ < 20 (corresponding to less than 0.1% of the image) were classified as incoherent. An example of a feature heat map and coherent regions extracted from the histogram is shown in Figure 5. In Figure 5A, feature values from a feature heat map are grouped into histogram bins. Figure 5B shows the original feature heat map and the extraction of coherent image regions assigned to Bin 4 of the histogram in Figure 5A.

The distance metric between two augmented histograms is typically based on the Bhattacharyya distance between histograms, weighted by the spatial similarity of the contents of bin *b* as in Birchfield and Rangarajan (2005). The difference between spatial histograms *h* and *h*′ is expressed as:

(3) $$\begin{array}{r} d\left(h,h^{\prime }\right)=\overset{\left|B\right|}{\underset{b=1}{\sum }}{\text{Ψ}}_{b}^{m}\left(1-\sqrt{n_{b}n_{b}^{\prime }}\right). \end{array}$$

The spatial weighting term ${\text{Ψ}}_{b}^{m}$ expresses the similarity of the *m* spatial regions in bin *b*. Previously, the Mahalanobis distance, or number of standard deviations between the means of the Gaussian distributions in each bin, has been used to weight the spatial similarity. In this work, we utilized the spatial weighting term to incorporate the point-wise similarity between corresponding points from two feature heat maps. This modification addressed both the need for spatial information and the loss of information due to histogram quantization. We defined the spatial weighting term as the mean feature error between histogram regions, normalized by the standard deviation at that point, calculated from the average model of asymmetry. This is expressed as:

(4) $$\begin{array}{r} {\text{Ψ}}_{b}^{m}=\overset{m}{\underset{i=1}{\sum }}w_{bi}\overset{k}{\underset{j=1}{\sum }}\frac{A\left(x_{j}\right)-A^{\prime }\left(x_{j}\right)}{\sigma_{j}}, \end{array}$$

where *w*_*bi*_ is the weight of the *ith* coherent region in bin *b*, *A*(*x*_*j*_) and $A^{\prime }\left(x_{j}\right)$ are the feature values from the two feature maps at the corresponding point *j* and $\sigma_{\bar{j}}$ is the standard deviation at point *j*. This spatial weighting term represents average error between feature maps, measured in standard deviations, for each coherent region. To achieve a symmetric distance measure, the total distance between *h* and *h*′ was defined as:

(5) $$\begin{array}{r} \rho \left(h,h^{\prime }\right)=\frac{\left(d\left(h,h^{\prime }\right)+d\left(h^{\prime },h\right)\right)}{2}. \end{array}$$

**Figure 6:**
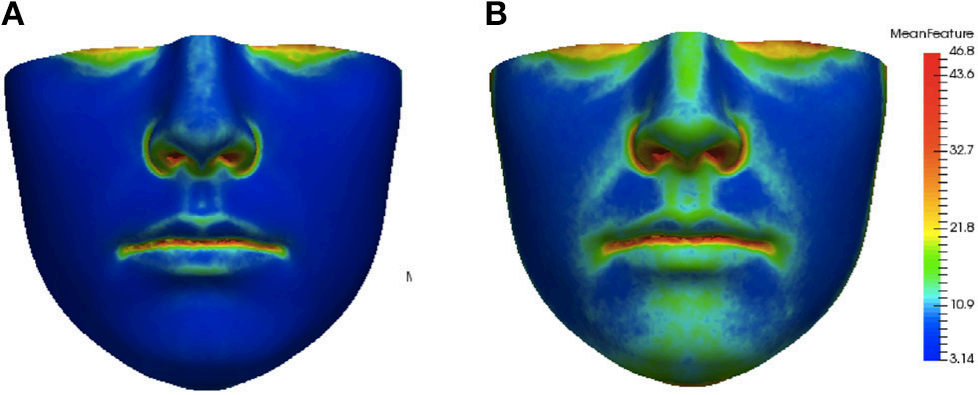
Group average heat maps from subjects with low asymmetry (angle of surface orientation score in lower 10 percent of data set) **(A)** and subjects with high asymmetry (angle of surface orientation score in upper 10 percent of data set) **(B)**. Regions with low asymmetry are blue and regions with high asymmetry are red.

This distance *ρ*(*h, h*′) was applied to assess the similarity of each individual feature heat map to the average feature heat map. This provided a hybrid local-plus-global summary of the abnormality of asymmetry of an individual and was assigned as our score of asymmetry. Average feature heat maps for subjects with the lowest (lower 10 percent of the data set) and highest (upper 10 percent of the data set) asymmetry scores for the angle of surface orientation feature are shown in Figure 6. In the average heat map from the high asymmetry score group in Figure 6B, regions with high values contributed the most to the score in individuals with high levels of asymmetry.

### 2.3. Genetic Association Analyses

Whole genome association with each phenotype score was done using PLINK (Purcell et al., 2007). SNPs with the minor allele present in less than 5 subjects were removed, resulting in 747,780 remaining SNPs. The first four principle components of the genetic data were used as covariates to adjust for the effects of ancestry. A linear model was used to test genetic association between our phenotype scores and each SNP, controlling for the effects of age and gender. The Benjamini–Hochberg procedure was used to adjust the original *p*-values globally over both phenotype scores in order to control the false discovery rate (FDR) (Benjamini and Hochberg, 1995). Genome-wide Complex Trait Analysis (GCTA) was used to estimate the proportion of variance in each phenotype score explained by all GWAS SNPs, i.e., heritability (Yang et al., 2011).

Each phenotype score was tested for associations with age and sex. The Pearson correlation coefficient was used to test for an association with age. An association with sex was tested using the Kendall rank correlation coefficient (tau). The Kendall test does not rely on the assumption of normally distributed data and so is more appropriate for dichotomous data such as sex. The correlations found between the asymmetry scores, age and sex were weak, though the correlations had high levels of significance in terms of the *p*-values, as reported in the Tables S2, S3. We speculate that this effect is likely due to the large sample size (i.e., statistical power), which made it possible to detect the significant associations.

## 3. Results

### 3.1. Angle of Surface Orientation Score

**Figure 7:**
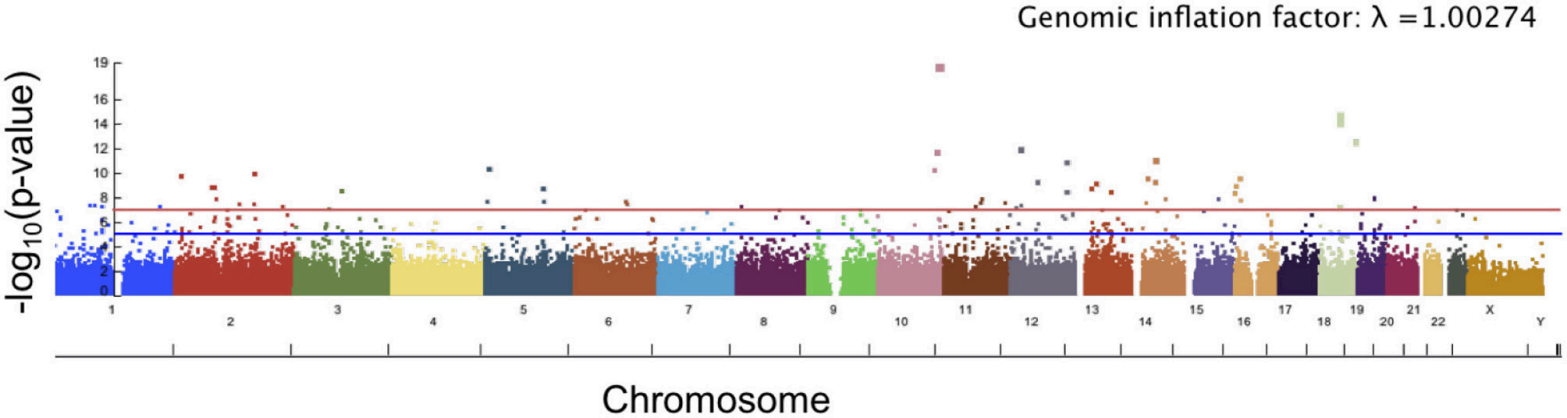
Manhattan plot showing significance of the relationship between each SNP and our angle of surface orientation score. The red line corresponds to a threshold of genome-wide significance of *p* = 5 × 10^−8^ and the blue line corresponds to a threshold of suggestive significance *p* = 1 × 10^−5^.

The top 10 SNPs significantly associated with the angle of surface orientation scores (*p*-value < 5 × 10^−8^) are listed in Table 1, and the Manhattan plot is shown in Figure 7. Of these SNPs with highly significant associations, three are located on genes with known links to craniofacial abnormality and asymmetry (*NFATC*1, *SOX*5, and *NBAS*) and one (*SNX*6) is on a gene with a potential link. *NFATC*1 encodes a transcription factor that plays a role in mandibular development and the Wnt signaling pathway, which is instrumental to facial morphogenesis (Winslow et al., 2006; Brugmann et al., 2007; Doraczynska-Kowalik et al., 2017). Mutations in *NFATC*1 are linked to Cherubism, a disorder characterized by abnormal bone tissue in the lower part of the face and a characteristic facial phenotype (Kadlub et al., 2016). A recent GWA study of morphological measurements has also suggested a possible link between this gene and measurements of the mouth (Lee et al., 2017). *SOX*5 encodes a transcription factor involved in the regulation of embryonic development that is thought to play a role in chondrogenesis. *SOX*5 is linked to Lamb-Shaffer Syndrome, which can cause an abnormal craniofacial phenotype including a facial asymmetry, depressed and/or broad nasal bridge, and bulbous nasal tip (Lamb et al., 2012). Mutations in *NBAS* are associated with Pelger-Huet Anomaly, which has a phenotype including facial asymmetry, long face, and straight nose (Segarra et al., 2015). It is also linked to Feingold Syndrome 1, which can result in craniofacial dismorphology including asymmetry, triangular shaped face, and flat nasal tip (Chen et al., 2012). *SNX*6, a member sorting nexin family, has not been definitively linked to craniofacial disorders, however multiple studies have suggested it as a candidate gene for holoprosencephaly, the most common developmental field defect in patterning of the human prosencephalon and associated craniofacial structures (Kamnasaran et al., 2005; Segawa et al., 2007). Also of interest is *TCF*7*L*1, which encodes for a transcription factor that mediates the Wnt signaling pathway and has been found to have high expression in the developing murine palate (Potter and Potter, 2015).

**Table 1:** Top 13 significant SNPs associated with angle of surface orientation scores.

**SNP**	**Chromosome**	**Gene**	***P*-values**	**FDR-corrected**
rs8088297	18	MAPK4	3.03 × 10^−15^	4.29 × 10^−10^
rs9953590	18	MAPK4	1.15 × 10^−14^	4.29 × 10^−10^
rs165149	18	**NFATC1**	3.76 × 10^−13^	1.63 × 10^−9^
rs4357783	12	**SOX5**	1.28 × 10^−12^	5.45 × 10^−9^
exm861196	10	TACC2	2.00 × 10^−12^	8.49 × 10^−9^
exm173678	2	**NBAS**	2.05 × 10^−10^	8.46 × 10^−7^
rs8006719	14	**SNX6**	3.24 × 10^−10^	1.33 × 10^−6^
rs7186843	16	TEKT5	3.81 × 10^−10^	1.56 × 10^−6^
rs4296170	14	TSPG3A	7.65 × 10^−10^	3.11 × 10^−6^
rs4597218	13	TGTF2F2	9.67 × 10^−10^	3.91 × 10^−6^
exm1214111	16	ZNF500	1.52 × 10^−9^	6.17 × 10^−6^
rs7563083	2	**TCF7L1**	1.39 × 10^−8^	5.35 × 10^−5^
rs8007933	14	SYNJ2BP	1.58 × 10^−8^	6.06 × 10^−5^

*Genes with known or potential association with craniofacial abnormality and asymmetry are boldfaced*.

The angle of surface orientation phenotype scores were assessed for heritability using GCTA and were found to have a proportion of variance consistent with a substantial heritability. Detailed results are reported in Table S4.

### 3.2. Angle of Deformation Score

**Figure 8:**
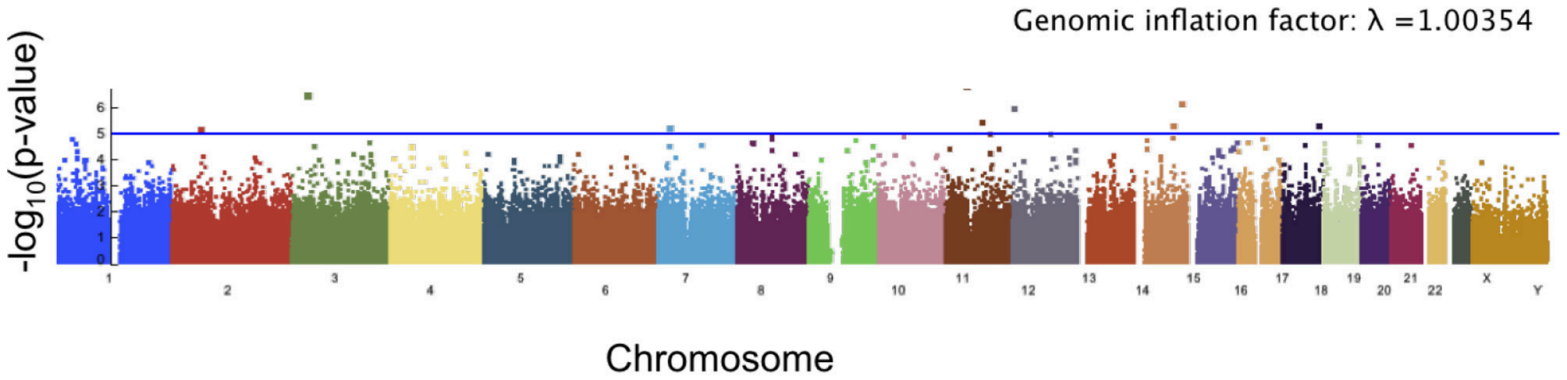
Manhattan plot showing significance of the relationship between each SNP and our angle of deformation scores. The blue line corresponds to a threshold of suggested significance *p* = 1 × 10^−5^.

The angle of deformation phenotype scores showed less significance than the facial asymmetry scores. The top 10 SNPs associated with the angle of deformation scores are reported in Table 2, and the Manhattan plot is shown in Figure 8. While many of the *p*-values for the SNPs associated with this phenotype score are not considered significant, it is possible that the multiple testing correction might have been overly conservative when significant linkage equilibrium was present. As there are a number of genes which have known or potential links to facial development or morphology, we reported the genes associated with these SNPs of interest, though the associations are weak.

**Table 2:** Top ten SNPs associated with angle of deformation scores.

**SNP**	**Chromosome**	**Gene**	***P*-value**	**FDR-corrected**
exm456223	5	GPBP1	8.21 × 10^−8^	2.95 × 10^−4^
exm903362	11	**AMBRA1**	1.53 × 10^−7^	5.33 × 10^−4^
rs17107396	14	**NRXN3**	5.11 × 10^−6^	1.35 × 10^−2^
exm1354641	17	TNRC6C	5.15 × 10^−6^	1.35 × 10^−2^
rs995266	14	**FANCC**	1.48 × 10^−5^	3.48 × 10^−2^
rs12596936	16	**FTO**	1.72 × 10^−5^	3.93 × 10^−2^
exm763253	9	**FANCC**	1.96 × 10^−5^	4.44 × 10^−2^
rs11928737	3	RARRES1	2.29 × 10^−5^	4.97 × 10^−2^
rs2276750	3	RARRES1	2.50 × 10^−5^	5.32 × 10^−2^
rs6588634	1	PRKAA2	2.76 × 10^−5^	5.78 × 10^−2^

*The genes with known or potential association with facial development or morphology are boldfaced*.

*AMBRA*1 encodes a protein that regulates different steps of the autophagic process and is an important regulator of embryonic development. Its mutation or inactivation in mice was shown to result in embryonic malformations (Fimia et al., 2007). Rare deletions in *NRXN*3 was linked to autism spectrum disorder (Vaags et al., 2012). While there is as yet no consensus on facial phenotypes associated with autism spectrum conditions (ASC), there is evidence to suggest that there are morphologically distinct subgroups within ASC that correspond with different cognitive and behavioral symptomatology (Boutrus et al., 2017). Two SNPs of interest are located on the gene *FANCC*, which encodes a DNA repair protein with a role in the maintenance of normal chromosome stability. FANCC is implicated in Gorlin syndrome that has a phenotype including broad nasal root, cleft lip, and cleft palate (Reichert et al., 2015). *FANCC* is also linked to Fanconi anemia that has a phenotype including craniosynostosis, microencephaly and small eyes (de Winter et al., 2000). *FTO* is a protein coding gene associated with growth retardation, developmental delay, and facial dysmorphism (Boissel et al., 2009; Daoud et al., 2015). The associated phenotype includes skull asymmetry, coarse facial features, abnormal positioning of the maxilla or mandible, prominent alveolar ridge, and cleft palate. The retinoid acid receptor-responsive gene *RARRES*1 contains two SNPs of interest. This gene is thought likely to play a role in embryonic morphogenesis (Oldridge et al., 2013).

The angle of deformation phenotype scores were assessed for heritiability using GCTA and were found to have a proportion of variance suggesting minimal heritiability and a *p*-value suggesting low significance. This is a possible explanation for the low levels of significance observed. These results are detailed in Figure S4.

### 3.3. Comparison to Asymmetry Scores Based on the Deformation Vector Magnitude

Angle-based measurements capture one aspect of asymmetry, which may be relevant to specific biological processes. Deformation magnitude, defined as the magnitude of the distance between each point on a facial image and its corresponding point on a mirrored image, is another common choice for mesh-based shape analysis. For comparison, we implemented our asymmetry score using the deformation magnitude as the local asymmetry feature. This local property was then used to calculate an overall score of asymmetry following the procedure outlined in the Methods section 2.2. The GWAS results from our magnitude-based asymmetry score are reported in Figure S3 and Table S5.

Since the average value of the deformation magnitude over an image surface is a metric frequently used in other studies, we also implemented an established measure from the literature to compare to our deformation magnitude asymmetry scores (Verhoeven et al., 2016). In this work, local asymmetry is defined as the magnitude of the distance between each point on a facial image and its corresponding point on a mirrored image. The measure of total facial asymmetry was calculated using the average of these distances over the face. This method was selected because it is similar to those used by several other groups and the results were validated on a data set with known ground truth. The GWAS results from this comparable deformable morphology approach are detailed in Figure S4.

Both magnitude-based methods we tested had lower significance and did not identify genes known to result in facial asymmetry. One gene of interest identified by both methods, *MYO*10, has been linked to craniofacial development in zebrafish. The genes identified by these two magnitude-only methods overlapped, but our magnitude-based asymmetry score showed higher levels of significance.

### 3.4. Comparison to Asymmetry Scores Based on Landmark Measurements

The motivation for developing the mesh-based methods in this work was to provide more complex phenotypes for genetic association than standard landmark-based approaches. Subtle differences in asymmetry that may be scientifically interesting are unlikely to be captured by landmark data, which is usually very sparse. While landmark-based methods may identify associations with genes of interest, they may identify different pathways than mesh-based analysis as they do not use data between the landmark points.

To compare our method to GWAS using a traditional, landmark-based approach of measuring asymmetry, a score of facial asymmetry was defined using the Procrustes distance between an image and its mirrored copy (Bookstein, 1997). Each image was rigidly aligned with its mirrored copy. A subset of the 12 bilaterally paired landmarks was selected from the original 18 landmarks shown in Figure S2 and the Euclidean distance between right/left landmark pairs was measured. The facial asymmetry score was calculated using the average of these distances. Using this method, no SNPs were found to meet the threshold of genome-wide significance of *p* = 5 × 10^−8^, as detailed in Figure S5. These results provide additional motivation for the use of mesh-based analysis, in addition to the improvements in precision and reproducibility,

## 4. Discussion

Asymmetry is the topic of a large number of studies investigating how genetic and environmental factors influence normal development. It is likely to be influenced by complex and interelated factors, which can be difficult to control for in human studies, presenting significant challenges for analysis. Asymmetry, especially fluctuating asymmetry, has been hypothesized to be closely linked to developmental instability and many studies have interpreted it as a marker of environmental stress during development (Klingenberg and McIntyre, 1998; DeLeon, 2007; Ozener, 2010). However, several recent studies have called these findings into question and have suggested a stronger role of heredity (Quinto-Sánchez et al., 2015, 2017). Further studies using genotype and phenotype data are needed to better understand how the developmental processes leading to asymmetry are impacted by environmental factors. While subjects in our study were screened for a number of possible environmental influences on facial asymmetry, as detailed in section 2.1, many other potential confounding factors remain, such as twinning status and smoking behavior that are unknown or could not feasibly be controlled for in this study. Despite these limitations, we have applied a data-driven approach to evaluate methods for quantifying aspects of asymmetry that may be related to biological processes resulting in facial asymmetry.

Consistent with other recent findings on the genetic basis of normal facial variation, several of the genes associated with variation in normal asymmetry are involved in syndromes with craniofacial phenotypes. This supports the hypothesis that common variants near the genes related to Mendelian syndromes are implicated in normal phenotypic variation (Shaffer et al., 2016). While we are cautious about interpreting the results from a angle of deformation asymmetry score, due to the weak associations, several of the genes of interest identified are associated with embryonic morphology and development and craniofacial abnormality. The genes identified by the angle of deformation score do not overlap with the genes identified by the angle of asymmetry score. This indicates the possibility that the two aspects of asymmetry quantified may be useful for identifying different biological pathways impacting facial asymmetry.

The heat maps of local asymmetry features provide information about the regions of the face that contribute most to the asymmetry scores. Figure 6B shows the average feature heat map for subjects with the highest asymmetry scores (top 10 percent of the data set). This heat map shows higher levels of asymmetry than the average feature heat map in Figure 4 and also suggests the relative importance of the nasal tip, nasal bridge, upper lip, and chin regions in subjects with high levels of normal asymmetry.

Questions still remain about the ability of complex phenotypes to be accurately associated with genetic data as the genotype-phenotype map for facial morphology is likely to be incredibly complex (Hallgrimsson et al., 2014). A single gene can result in local or global shape differences and be intertwined with environmental factors. Despite these challenges, we have demonstrated that our hybrid local-to-global score of abnormal asymmetry was able to find associations with genes known to play a role in craniofacial morphology and asymmetry. While we do not have an assurance that our automated phenotyping method is the optimal strategy to summarize phenotypes for genetic association, the significance of the results motivates its further development. One limitation of this study was our lack of a comparable dataset with which to replicate our findings. If one becomes available in the future, applying these methods to identify an overlapping set of genes would significantly strengthen the findings in this work.

In future work, new local morphological metrics can be investigated using this framework. This method can also be implemented to compare subjects to an average model of a group of interest, rather than a control population, to assess similarity to a known phenotype. Taking a data-driven approach to optimizing phenotypic descriptors, guided by the significance of the genetic associations uncovered, will contribute to both our understanding of the genetic basis of human facial variation and the creation of new metrics for biologically relevant phenotype data.

## Ethics Statement

The study was carried out in accordance with the recommendations of University of Washington IRB #42874 with written informed consent from all subjects. All subjects gave written informed consent in accordance with the Declaration of Helsinki. The University of Washington IRB approved the protocol.

## Data Availability Statement

The datasets analyzed for this study were obtained from FaceBase (www.facebase.org), and were generated by projects U01DE020078 and U01DE020054. The FaceBase Data Management Hub (U01DE020057) and the FaceBase Consortium are funded by the National Institute of Dental and Craniofacial Research. The phenotype scores developed for this work will be made available on request.

## Author Contributions

SR carried out the main efforts on the research including developing the theory behind the angle of surface orientation score and the angle of deformation score as well also carrying out all the experiments, and writing the paper. S-IL acted as a consultant on the analysis of the GWAS results and helped to write the Results and Discussion sections of the paper. LS served as the primary adviser to SR in this work.

### Conflict of Interest Statement

The authors declare that the research was conducted in the absence of any commercial or financial relationships that could be construed as a potential conflict of interest.

## References

[B1] Benjamini Y. Hochberg Y. (1995). Controlling the false discovery rate: a practical and powerful approach to multiple testing. J. R. Stat. Soc. Ser. B (Methodol.). 57, 289–300. 10.1111/j.2517-6161.1995.tb02031.x

[B2] Birchfield S. T. Rangarajan S. (2005). Spatiograms versus histograms for region-based tracking in Computer Vision and Pattern Recognition, 2005. CVPR 2005. IEEE Computer Society Conference on, Vol. 2 (San Diego, CA: IEEE), 1158–1163. 10.1109/CVPR.2005.330

[B3] Boissel S. Reish O. Proulx K. Kawagoe-Takaki H. Sedgwick B. Yeo G. S.. (2009). Loss-of-function mutation in the dioxygenase-encoding fto gene causes severe growth retardation and multiple malformations. Am. J. Hum. Genet. 85, 106–111. 10.1016/j.ajhg.2009.06.002 19559399 PMC2706958

[B4] Bookstein F. L. (1997). Morphometric Tools for Landmark Data: Geometry and Biology. Cambridge: Cambridge University Press.

[B5] Boutrus M. Maybery M. T. Alvares G. A. Tan D. W. Varcin K. J. Whitehouse A. J. (2017). Investigating facial phenotype in autism spectrum conditions: the importance of a hypothesis driven approach. Autism Res. 10, 1910–1918. 10.1002/aur.1824 28816000

[B6] Brugmann S. A. Goodnough L. H. Gregorieff A. Leucht P. ten Berge D. Fuerer C.. (2007). Wnt signaling mediates regional specification in the vertebrate face. Development 134, 3283–3295. 10.1242/dev.005132 17699607

[B7] Chen C.-P. Lin S.-P. Chern S.-R. Wu P.-S. Chang S.-D. Ng S.-H.. (2012). A de novo 4.4-mb microdeletion 2p24. 3 - p24. 2 in a girl with bilateral hearing impairment, microcephaly, digit abnormalities and feingold syndrome. Eur. J. Med. Genet. 55, 666–669. 10.1016/j.ejmg.2012.07.003 22842076

[B8] Claes P. Liberton D. K. Daniels K. Rosana K. M. Quillen E. E. Pearson L. N. (2014). Modeling 3d facial shape from dna. PLoS Genet. 10:e1004224 10.1371/journal.pgen.1004224 24651127 PMC3961191

[B9] Claes P. Walters M. Vandermeulen D. Clement J. G. (2011). Spatially-dense 3d facial asymmetry assessment in both typical and disordered growth. J. Anat. 219, 444–455. 10.1111/j.1469-7580.2011.01411.x 21740426 PMC3187867

[B10] Daoud H. Zhang D. McMurray F. Yu A. Luco S. M. Vanstone J.. (2015). Identification of a pathogenic fto mutation by next-generation sequencing in a newborn with growth retardation and developmental delay. J. Med. Genet. 53, 200–207. 10.1136/jmedgenet-2015-103399 26378117

[B11] Darvann T. A. Hermann N. V. Demant S. Larsen P. Ólafsdóttir H. Thorup S. S. (2011). Automated quantification and analysis of facial asymmetry in children with arthritis in the temporomandibular joint in Biomedical Imaging: From Nano to Macro, 2011 IEEE International Symposium on (Chicago, IL: IEEE), 1193–1196.

[B12] de Winter J. P. Léveillé F. van Berkel C. G. Rooimans M. A. van der Weel L. Steltenpool J.. (2000). Isolation of a cdna representing the fanconi anemia complementation group e gene. Am. J. Hum. Genet. 67, 1306–1308. 10.1016/S0002-9297(07)62959-0 11001585 PMC1288571

[B13] DeLeon V. B. (2007). Fluctuating asymmetry and stress in a medieval nubian population. Am. J. Phys. Anthropol. 132, 520–534. 10.1002/ajpa.20549 17243154

[B14] Demant S. Hermann N. V. Darvann T. A. Zak M. Schatz H. Larsen P.. (2010). 3d analysis of facial asymmetry in subjects with juvenile idiopathic arthritis. Rheumatology 50, 586–592. 10.1093/rheumatology/keq329 21097878

[B15] Devlin M. F. Ray A. Raine P. Bowman A. Ayoub A. F. (2007). Facial symmetry in unilateral cleft lip and palate following alar base augmentation with bone graft: a three-dimensional assessment. Cleft Palate-Craniofacial J. 44, 391–395. 10.1597/06-179.1 17608557

[B16] Dindaroğlu F. Kutlu P. Duran G. S. Görgülü S. Aslan E. (2016). Accuracy and reliability of 3d stereophotogrammetry: a comparison to direct anthropometry and 2d photogrammetry. Angle Orthodont. 86, 487–494. 10.2319/041415-244.1 26267357 PMC8601748

[B17] Djordjevic J. Lewis B. M. Donaghy C. E. Zhurov A. I. Knox J. Hunter L.. (2012). Facial shape and asymmetry in 5-year-old children with repaired unilateral cleft lip and/or palate: an exploratory study using laser scanning. Eur. J. Orthodont. 36, 497–505. 10.1093/ejo/cjs075 23041935

[B18] Doraczynska-Kowalik A. Nelke K. H. Pawlak W. Sasiadek M. M. Gerber H. (2017). Genetic factors involved in mandibular prognathism. J. Craniof. Surg. 28, e422–e431. 10.1097/SCS.0000000000003627 28570402

[B19] Fimia G. M. Stoykova A. Romagnoli A. Giunta L. Di Bartolomeo S. Nardacci R.. (2007). Ambra1 regulates autophagy and development of the nervous system. Nature 447, 1121–1125. 10.1038/nature05925 17589504

[B20] Hallgrimsson B. Mio W. Marcucio R. S. Spritz R. (2014). Let's face it—complex traits are just not that simple. PLoS Genet. 10:e1004724 10.1371/journal.pgen.1004724 25375250 PMC4222688

[B21] Hutton T. J. Buxton B. F. Hammond P. Potts H. W. (2003). Estimating average growth trajectories in shape-space using kernel smoothing. IEEE Trans. Med. Imaging 22, 747–753. 10.1109/TMI.2003.814784 12872950

[B22] Kadlub N. Sessiecq Q. Dainese L. Joly A. Lehalle D. Marlin S.. (2016). Defining a new aggressiveness classification and using nfatc1 localization as a prognostic factor in cherubism. Hum. Pathol. 58, 62–71. 10.1016/j.humpath.2016.07.019 27498064

[B23] Kamnasaran D. Chen C.-P. Devriendt K. Mehta L. Cox D. W. (2005). Defining a holoprosencephaly locus on human chromosome 14q13 and characterization of potential candidate genes. Genomics 85, 608–621. 10.1016/j.ygeno.2005.01.010 15820313

[B24] Klingenberg C. P. McIntyre G. S. (1998). Geometric morphometrics of developmental instability: analyzing patterns of fluctuating asymmetry with procrustes methods. Evolution 52, 1363–1375. 10.1111/j.1558-5646.1998.tb02018.x 28565401

[B25] Kornreich D. Mitchell A. A. Webb B. D. Cristian I. Jabs E. W. (2016). Quantitative assessment of facial asymmetry using three-dimensional surface imaging in adults: validating the precision and repeatability of a global approach. Cleft Palate Craniofac J. 53, 126–131. 10.1597/13-353 25489769

[B26] Lamb A. N. Rosenfeld J. A. Neill N. J. Talkowski M. E. Blumenthal I. Girirajan S.. (2012). Haploinsufficiency of sox5 at 12p12. 1 is associated with developmental delays with prominent language delay, behavior problems, and mild dysmorphic features. Hum. Mutat. 33, 728–740. 10.1002/humu.22037 22290657 PMC3618980

[B27] Lanche S. Darvann T. Ólafsdóttir H. (2007). A statistical model of head asymmetry in infants with deformational plagiocephaly. Image Anal. 4522, 898–907. 10.1007/978-3-540-73040-8_91

[B28] Lee M. K. Shaffer J. R. Leslie E. J. Orlova E. Carlson J. C. Feingold E.. (2017). Genome-wide association study of facial morphology reveals novel associations with frem1 and park2. PLoS ONE 12:e0176566. 10.1371/journal.pone.0176566 28441456 PMC5404842

[B29] Linden O. E. Taylor H. O. Vasudavan S. Byrne M. E. Deutsch C. K. Mulliken J. B.. (2017). Three-dimensional analysis of nasal symmetry following primary correction of unilateral cleft lip nasal deformity. Cleft Palate-Craniofacial J. 54, 715–719. 10.1597/16-073 27441702

[B30] Lyons D. M. (2009). Sharing landmark information using mixture of gaussian terrain spatiograms in Intelligent Robots and Systems, 2009. IROS 2009. IEEE/RSJ International Conference on (St. Louis, MO: IEEE), 5603–5608. 10.1109/IROS.2009.5354223

[B31] Mercan E. Oestreich M. Fisher D. M. Allori A. C. Beals S. P. Samson T. D.. (2018). Objective assessment of the unilateral cleft lip nasal deformity using three-dimensional stereophotogrammetry: Severity and outcome. Plastic Reconstruct. Surg. 141, 547e–558e. 10.1097/PRS.0000000000004233 29257001 PMC5876085

[B32] Mercan E. Shapiro L. G. Weinberg S. M. Lee S.-I. (2013). The use of pseudo-landmarks for craniofacial analysis: a comparative study with l 1-regularized logistic regression in Engineering in Medicine and Biology Society (EMBC), 2013 35th Annual International Conference of the IEEE (Osaka: IEEE), 6083–6086. 10.1109/EMBC.2013.6610940 PMC3836675 24111127

[B33] Oldridge E. E. Walker H. Stower M. Simms M. Mann V. Collins A.. (2013). Retinoic acid represses invasion and stem cell phenotype by induction of the metastasis suppressors rarres1 and lxn. Oncogenesis 2:e45. 10.1038/oncsis.2013.6 23588494 PMC3641360

[B34] Öwall L. Darvann T. A. Larsen P. Hove H. D. Hermann N. V. Bøgeskov L.. (2016). Facial asymmetry in children with unicoronal synostosis who have undergone craniofacial reconstruction in infancy. Cleft Palate-Craniofacial J. 53, 385–393. 10.1597/15-089 26418148

[B35] Ozener B. (2010). Fluctuating and directional asymmetry in young human males: effect of heavy working condition and socioeconomic status. Am. J. Phys. Anthropol. 143, 112–120. 10.1002/ajpa.21300 20734438

[B36] Potter A. S. Potter S. S. (2015). Molecular anatomy of palate development. PLoS ONE 10:e0132662. 10.1371/journal.pone.0132662 26168040 PMC4500583

[B37] Prabhu J. Kumar J. S. (2014). Wavelet based content based image retrieval using color and texture feature extraction by gray level coocurence matrix and color coocurence matrix. J. Comput. Sci. 10:15 10.3844/jcssp.2014.15.22

[B38] Purcell S. Neale B. Todd-Brown K. Thomas L. Ferreira M. A. Bender D.. (2007). Plink: a tool set for whole-genome association and population-based linkage analyses. Am. J. Hum. Genet. 81, 559–575. 10.1086/519795 17701901 PMC1950838

[B39] Quinto-Sánchez M. Adhikari K. Acuña-Alonzo V. Cintas C. Silva de Cerqueira C. C. Ramallo V. (2015). Facial asymmetry and genetic ancestry in l atin a merican admixed populations. Am. J. Phys. Anthropol. 157, 58–70. 10.1002/ajpa.22688 25582401

[B40] Quinto-Sánchez M. Cintas C. de Cerqueira C. C. S. Ramallo V. Acuña-Alonzo V. Adhikari K. (2017). Socioeconomic status is not related with facial fluctuating asymmetry: evidence from latin-american populations. PLoS ONE 12:e0169287 10.1371/journal.pone.0169287 28060876 PMC5218465

[B41] Reichert S. C. Zelley K. Nichols K. E. Eberhard M. Zackai E. H. Martinez-Poyer J. (2015). Diagnosis of 9q22. 3 microdeletion syndrome *in utero* following identification of craniosynostosis, overgrowth, and skeletal anomalies. Am. J. Med. Genet. A 167, 862–865. 10.1002/ajmg.a.37013 25706929

[B42] Rolfe S. Shapiro L. Cox T. Maga A. Cox L. (2011). A landmark-free framework for the detection and description of shape differences in embryos in Engineering in Medicine and Biology Society, EMBC, 2011 Annual International Conference of the IEEE (Boston, MA: IEEE), 5153–5156. 10.1109/IEMBS.2011.6091276 PMC3261520 22255499

[B43] Rolfe S. M. Camci E. D. Mercan E. Shapiro L. G. Cox T. C. (2013). A new tool for quantifying and characterizing asymmetry in bilaterally paired structures in Engineering in Medicine and Biology Society (EMBC), 2013 35th Annual International Conference of the IEEE (Osaka: IEEE), 2364–2367. 10.1109/EMBC.2013.6610013 PMC3801450 24110200

[B44] Rolfe S. M. Cox L. Shapiro L. G. Cox T. C. (2014). A new landmark-independent tool for quantifying and characterizing morphologic variation in International Conference Image Analysis and Recognition (Vilamoura: Springer), 75–83.

[B45] Segarra N. G. Ballhausen D. Crawford H. Perreau M. Campos-Xavier B. van Spaendonck-Zwarts K. (2015). Nbas mutations cause a multisystem disorder involving bone, connective tissue, liver, immune system, and retina. Am. J. Med. Genet. A 167, 2902–2912. 10.1002/ajmg.a.37338 26286438

[B46] Segawa Y. Itokazu N. Hirose A. Nakagawa S. Takashima S. (2007). A case of partial 14q-with facial features of holoprosencephaly and hydranencephaly. Pediatr. Neurol. 37, 51–54. 10.1016/j.pediatrneurol.2007.02.010 17628223

[B47] Shaffer J. R. Orlova E. Lee M. K. Leslie E. J. Raffensperger Z. D. Heike C. L.. (2016). Genome-wide association study reveals multiple loci influencing normal human facial morphology. PLoS Genet. 12:e1006149. 10.1371/journal.pgen.1006149 27560520 PMC4999139

[B48] Stauber I. Vairaktaris E. Holst A. Schuster M. Hirschfelder U. Neukam F. W.. (2008). Three-dimensional analysis of facial symmetry in cleft lip and palate patients using optical surface data. J. Orof. Orthoped. 69, 268–282. 10.1007/s00056-008-0746-1 18797831

[B49] Vaags A. K. Lionel A. C. Sato D. Goodenberger M. Stein Q. P. Curran S.. (2012). Rare deletions at the neurexin 3 locus in autism spectrum disorder. Am. J. Hum. Genet. 90, 133–141. 10.1016/j.ajhg.2011.11.025 22209245 PMC3257896

[B50] Verhoeven T. Xi T. Schreurs R. Bergé S. Maal T. (2016). Quantification of facial asymmetry: a comparative study of landmark-based and surface-based registrations. J. Cranio-Maxillofacial Surg. 44, 1131–1136. 10.1016/j.jcms.2016.07.017 27519663

[B51] Weinberg S. M. Raffensperger Z. D. Kesterke M. J. Heike C. L. Cunningham M. L. Hecht J. T.. (2016). The 3d facial norms database: Part 1. a web-based craniofacial anthropometric and image repository for the clinical and research community. Cleft Palate-Craniofacial J. 53, 185–197. 10.1597/15-199 26492185 PMC4841760

[B52] Windhager S. Schaschl H. Schaefer K. Mitteroecker P. Huber S. Wallner B. (2014). Variation at genes influencing facial morphology are not associated with developmental imprecision in human faces. PLoS ONE 9:e99009 10.1371/journal.pone.0099009 24914781 PMC4051657

[B53] Winslow M. M. Pan M. Starbuck M. Gallo E. M. Deng L. Karsenty G.. (2006). Calcineurin/nfat signaling in osteoblasts regulates bone mass. Dev. Cell 10, 771–782. 10.1016/j.devcel.2006.04.006 16740479

[B54] Wu J. Tse R. Shapiro L. G. (2014). Automated face extraction and normalization of 3d mesh data in Engineering in Medicine and Biology Society (EMBC), 2014 36th Annual International Conference of the IEEE (Chicago, IL: IEEE), 750–753. 10.1109/EMBC.2014.6943699 PMC4287986 25570067

[B55] Yang J. Lee S. H. Goddard M. E. Visscher P. M. (2011). Gcta: a tool for genome-wide complex trait analysis. Am. J. Hum. Genet. 88, 76–82. 10.1016/j.ajhg.2010.11.011 21167468 PMC3014363

[B56] Yu Z. Mu X. Feng S. Han J. Chang T. (2009). Flip-registration procedure of three-dimensional laser surface scanning images on quantitative evaluation of facial asymmetries. J. Craniof. Surg. 20, 157–160. 10.1097/SCS.0b013e318191ce88 19165015

[B57] Zeng M. Wu Z. Tian C. Zhang L. Hu L. (2015). Efficient person re-identification by hybrid spatiogram and covariance descriptor in Proceedings of the IEEE Conference on Computer Vision and Pattern Recognition Workshops (Boston, MA), 48–56.

[B58] Zhao Y.-J. Xiong Y.-X. Wang Y. (2017). Three-dimensional accuracy of facial scan for facial deformities in clinics: a new evaluation method for facial scanner accuracy. PLoS ONE 12:e0169402. 10.1371/journal.pone.0169402 28056044 PMC5215889

