# Supplementary material for: Associations Between Genetic Data and Quantitative Assessment of Normal Facial Asymmetry
Source: Front Genet. 2018 Dec 18;9:659. doi: 10.3389/fgene.2018.00659 (PMC6315129; doi:10.3389/fgene.2018.00659)
Supplement: Supplementary file 1 [file Data_Sheet_1.PDF]

Supplementary Material: Associations Between Genetic Data and Quantitative Assessment of Normal Facial Asymmetry

1 GWAS EXCLUDING SUBJECTS YOUNGER THAN 14

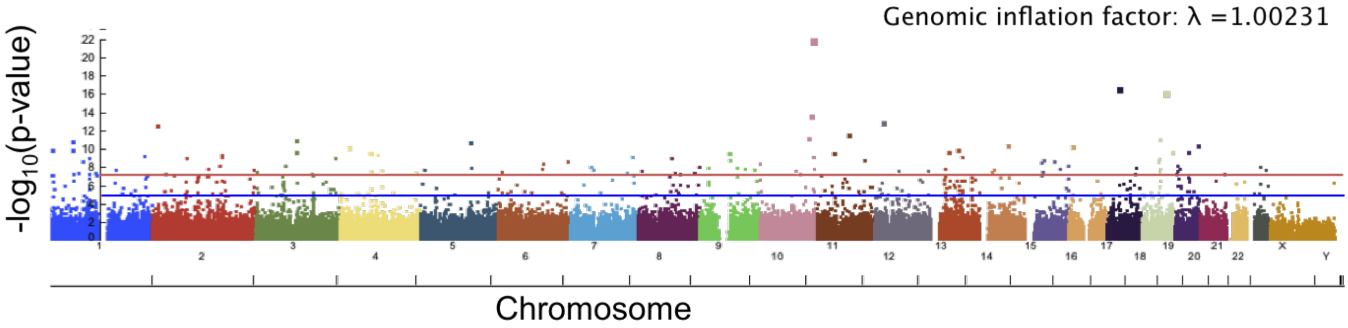

Figure S1. Manhattan plot showing significance of the relationship between each SNP and our asymmetry score using the surface orientation score. The red line corresponds to a threshold of genome-wide significance of $p = 5 \times 10^{-8}$ and the blue line corresponds to a threshold of suggested significance $p = 1 \times 10^{-5}$.

Table S1. Top significant SNPs associated with our asymmetry scores using the surface orientation feature after exclusion of subjects younger than 14 years old.

SNP	Chromosome	Gene	P-Values	FDR-corrected
exm861196	10	TACC2	3.12×10^{-14}	3.50×10^{-10}
rs4357783	12	SOX5	1.43×10^{-13}	3.74×10^{-10}
exm173678	2	NAG	3.47×10^{-13}	7.53×10^{-10}
rs8088297	18	MAPK4	1.01×10^{-11}	2.12×10^{-8}
rs7186843	16	TEKT5	7.31×10^{-11}	1.50×10^{-7}
exm20439	1	FAM131C	1.57×10^{-10}	3.19×10^{-7}
exm20576	1	FAM131C	1.57×10^{-10}	3.19×10^{-7}
rs11208297	1	ROR1	1.62×10^{-10}	3.29×10^{-7}
rs165149	18	NFATC1	2.52×10^{-10}	5.09×10^{-7}
rs4597218	13	GTF2F2	2.93×10^{-10}	5.89×10^{-7}

2 FACIAL SURFACE LANDMARKS

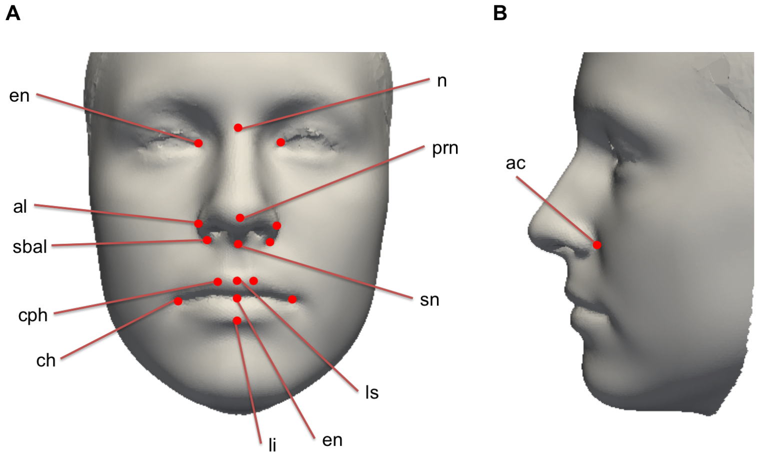

Figure S2. Facial mesh showing 18 landmarks used for alignment and analysis. Landmarks shown in the frontal view (A) are: endocanthion (en); alare (al); subalare (sbal); crista philtra (cph); chelion (ch); nasion (n), pronasale (prn), subnasale (sn); nasion; pronasale (prn); subnasale (sn); labiale superius (ls); labiale inferius (li). In this view, bilateral landmarks are labeled only on the right side. The lateral view (B) shows the landmark alar curvature point (ac). This is a bilateral point shown only on the left side.

3 CORRELATION OF ASYMMETRY SCORES WITH AGE

Table S2. Pearson correlation coefficient and significance of correlation between age and each asymmetry score

Phenotype score	Pearson correlation coefficient	P-Value
Angle of surface orientation	0.1803	1.08×10^{-18}
Angle of deformation	0.1146	2.40×10^{-8}

4 CORRELATION OF ASYMMETRY SCORES WITH SEX

Table S3. Kendall's tau coefficient and significance of correlation between sex and each asymmetry score

Phenotype score	Kendall's tau coefficient	P-Value
Angle of surface orientation	-0.0747	8.94×10^{-6}
Angle of deformation	-0.0441	0.0088

5 HERITABILITY OF ASYMMETRY SCORES

Table S4. Proportion of variance for each asymmetry score explained by all GWAS SNPs, estimated using Genome-wide Complex Trait Analysis

Phenotype score	Proportion of variance	Standard Error	P-Value
Angle of surface orientation	0.5688	0.1486	5.13×10^{-5}
Angle of deformation	1.00×10^{-6}	0.1453	0.5

6 OUR ASYMMETRY SCORE USING DEFORMATION MAGNITUDE FEATURE

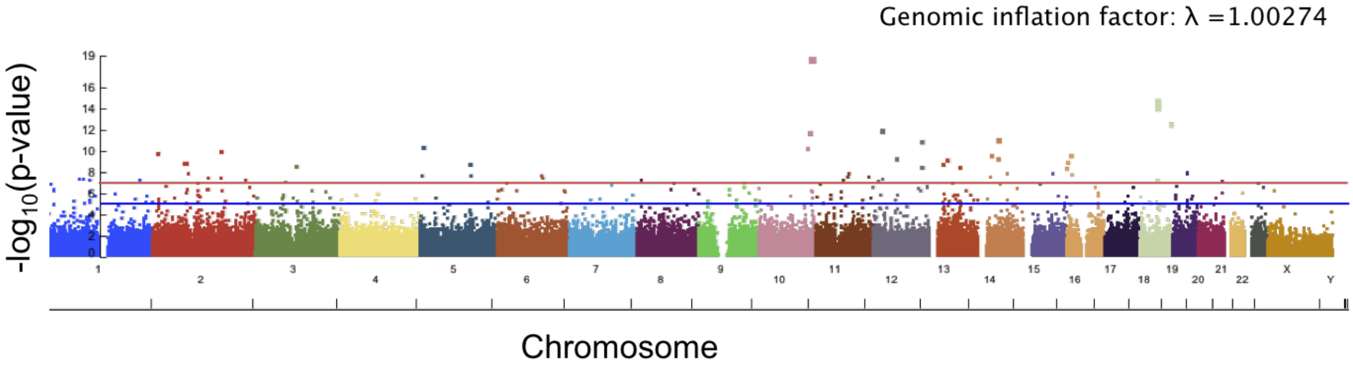

Figure S3. Manhattan plot showing significance of the relationship between each SNP and our asymmetry score using the deformation magnitude feature. This local feature is defined at each point on an individual facial mesh as the length vector between that point and the corresponding point on the mirror image. This local property is then used to calculate an overall score of asymmetry following the procedure outlined in the Methods section 2.2. The red line corresponds to a threshold of genome-wide significance of $p = 5 \times 10^{-8}$ and the blue line corresponds to a threshold of suggested significance $p = 1 \times 10^{-5}$.

Table S5. Top significant SNPs associated with our asymmetry score using deformation magnitude feature.

SNP	Chromosome	Gene	P-Values	FDR-corrected
rs1872004	5	MYO10	1.71×10^{-6}	0.0034
exm1347199	17	ABCA5	3.15×10^{-6}	0.0063
rs6504459	17	ABCA5	3.15×10^{-6}	0.0063
exm446558	5	MYO10	3.95×10^{-6}	.0078
rs10955278	8	CNBD1	4.25×10^{-6}	0.0084
rs2905419	8	CNBD1	4.38×10^{-6}	0.0086
rs3756766	5	CDC25C	4.80×10^{-6}	0.0094
rs7717298	5	MYO10	5.00×10^{-6}	0.0098
rs10900851	5	CDC25C	5.81×10^{-6}	0.0113
exm2257834	6	CYP39A1	6.65×10^{-6}	0.0127

7 GWAS USING COMPARABLE DEFORMABLE MORPHOLOGY APPROACH

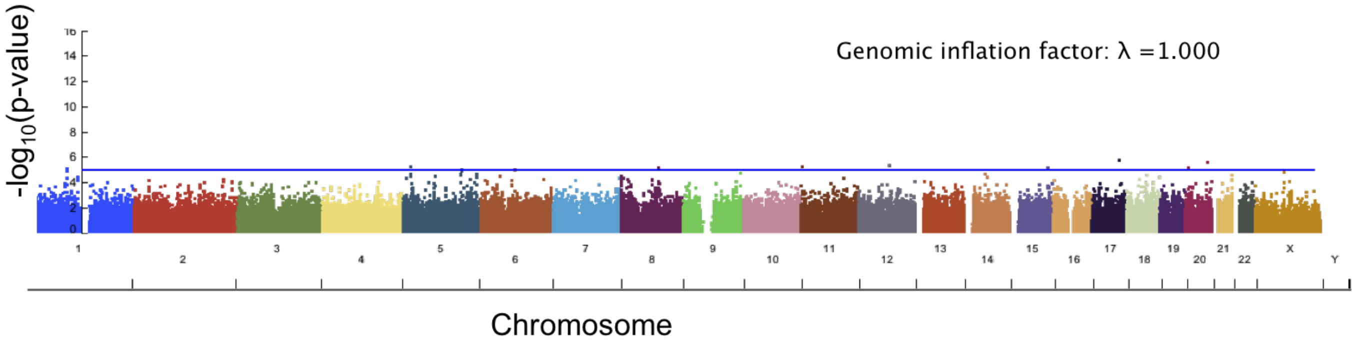

Figure S4. Manhattan plot showing significance of the relationship between each SNP and a score assigned by a comparable deformable morphology method as described in (Verhoeven, 2016). In this work, a local asymmetry feature is defined as the magnitude of the distance between each point on a facial image and its corresponding point on a mirrored image. A measure of total facial asymmetry was calculated using the average of these distances over the face. The blue line corresponds to a threshold of suggested significance $p = 1 \times 10^{-5}$. No SNPs were found to meet the threshold of genome-wide significance of $p = 5 \times 10^{-8}$

8 GWAS USING TRADITIONAL LANDMARK-BASED APPROACH

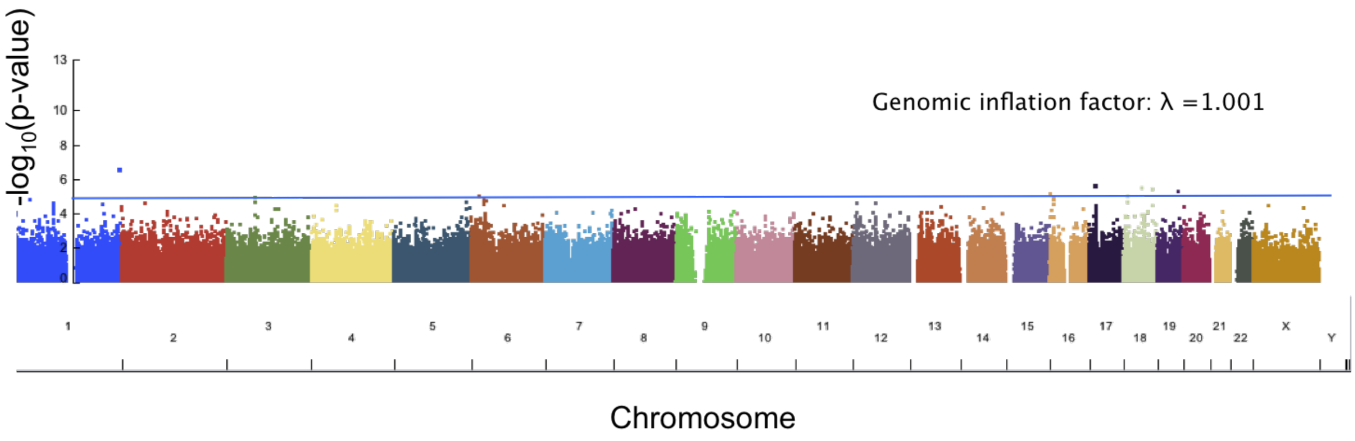

Figure S5. Manhattan plot showing significance of the relationship between each SNP and a score assigned by a traditional landmark-based method that uses the Procrustes distance between right and left sides (Bookstein, 1997). Images were aligned with a mirrored copy and the Euclidean distance between 12 right/left landmark pairs was measured. A score of total facial asymmetry was calculated using the average of these distances. The blue line corresponds to a threshold of suggested significance $p = 1 \times 10^{-5}$. No SNPs were found to meet the threshold of genome-wide significance of $p = 5 \times 10^{-8}$
